# Biosensor Based on Tyrosinase Immobilized on Graphene-Decorated Gold Nanoparticle/Chitosan for Phenolic Detection in Aqueous

**DOI:** 10.3390/s17051132

**Published:** 2017-05-16

**Authors:** Fuzi Mohamed Fartas, Jaafar Abdullah, Nor Azah Yusof, Yusran Sulaiman, Mohd Izham Saiman

**Affiliations:** 1 Department of Chemistry, Faculty of Science, Universiti Putra Malaysia, 43400 UPM Serdang, Selangor D.E., Malaysia; fuzi2007@gmail.com (F.M.F.); azahy@upm.edu.my (N.A.Y.); yusran@upm.edu.my (Y.S.); mohdizham@upm.edu.my (M.I.S.); 2 Institute of Advanced Technology, Universiti Putra Malaysia, 43400 UPM Serdang, Selangor D.E., Malaysia

**Keywords:** tyrosinase, graphene-gold nanocomposite, electrochemical biosensor, phenolic compounds

## Abstract

In this research work, electrochemical biosensor was fabricated based on immobilization of tyrosinase onto graphene-decorated gold nanoparticle/chitosan (Gr-Au-Chit/Tyr) nanocomposite-modified screen-printed carbon electrode (SPCE) for the detection of phenolic compounds. The nanocomposite film was constructed via solution casting method. The electrocatalytic activity of the proposed biosensor for phenol detection was studied using differential pulse voltammetry (DPV) and cyclic voltammetry (CV). Experimental parameters such as pH buffer, enzyme concentration, ratio of Gr-Au-Chit, accumulation time and potential were optimized. The biosensor shows linearity towards phenol in the concentration range from 0.05 to 15 μM with sensitivity of 0.624 μA/μM and the limit of detection (LOD) of 0.016 μM (S/N = 3). The proposed sensor also depicts good reproducibility, selectivity and stability for at least one month. The biosensor was compared with high-performance liquid chromatography (HPLC) method for the detection of phenol spiked in real water samples and the result is in good agreement and comparable.

## 1. Introduction

Phenol and substituted phenols are toxic chemicals that are released into the environment from industrial wastewater such as textile, pesticides, mining, dyes, and petrochemical and pharmaceutical industries. These classes of compounds reach into the food chain by wastewaters, then lead to hazardous and toxic effects on aquatic organisms [1]. It is reported that more than 165 phenols have an environmental toxic effect. It had also been reported to have adverse health effects on human organs such as the lungs, liver, kidneys, respiratory tract, and genito-urinary systems [2]. Due to their inherent toxicity, these kinds of compounds are claimed as priority hazardous toxic substances by the Toxic Substances and Disease Registry Agency. Thus, the development of simple techniques for the detection of trace amounts of these compounds is still a challenge and important due to their serious toxicity to the environment and human health. Existing methods for detecting phenolic compounds in water samples are based on classical techniques, for example: spectrophotometry, gas chromatography and high-performance liquid chromatography (HPLC) [3], which are tedious procedures, require experienced technicians to operate, time-consuming and involve expensive instrumentation. Therefore, there is an urgent need to develop a simple, rapid, sensitive, selective, user-friendly and less expensive method for the detection of phenolic derivatives in water systems.

Electrochemical technique studies of redox behaviour of different compounds in chemical reactions usually offer high sensitivity, good selectivity, rapid response and low cost. Electrochemical biosensors are considered a promising method for phenolic compounds detection, based on immobilization of bioreceptor on the electrode surface [4,5]. In biosensor development, immobilization is a key, important step, which involves application of novel sensing material with good electronic properties, and is biocompatible, stable, easily accessible by the analyte, and has a large surface area. In recent years, common methods explored for protein immobilization include physical adsorption [6], sol-gel encapsulation [7], electrochemical entrapment within a polymer or a composite matrix [8], incorporation within carbon paste [9], and covalent cross-linking [10], respectively.

Graphene has ideal and unique properties such as excellent conductivity, large surface area, good chemical stability, mechanical strength and high charge transport mobility [11]. Therefore, it has attracted interest significantly in several technological application fields including nanoelectronics, nanocomposite, biosensing and bioelectronics [12]. In a particular application, graphene requires chemical functionalization to meet the desired condition, which sometimes causes significant decreases in the electrical conductivity of the functionalized graphene [13]. This may be due to the increasing of the defect concentration and the doping of insulating materials on the graphene surface, which will deteriorate the electrons transfer and affect the electrical conductivity. Thus, there are still needs for studying compositing graphene with other nanomaterials, which can achieve graphene surface modification to enhance conductivity.

Graphene nanosheets have been used for anchoring noble metal nanoparticles using several environmentally friendly methods such as electrodeposition [14], assembly of metal nanoparticles on functionalized graphene surface [15] and microwave treatment [16]. For example, Yang et al. [17] anchored gold nanoparticles (AuNPs) onto carboxylic graphene nanosheets by using green method without adding any reductant or protecting molecule. Tang et al. [18] reported the electrodeposition of reduced graphene oxide and gold nanoparticles onto the surface of a glassy carbon electrode (GCE) without involving a toxic reductant. The rationale behind this choice of nanomaterial is based on the fact that AuNPs provide a favorable environment for enzyme immobilization and to facilitate electron transfer between the immobilized enzyme and the electrode [19,20]. The large surface area of graphene along with electronic properties and good mechanical strength make them a promising support material for metal nanoparticles. These unique criteria decrease the agglomeration of AuNPs and enhance their stability into graphene nanosheet matrix, therefore, the potential application in biosensing has been explored.

Recently, applications of graphene nanocomposite have attracted growing interest in many research areas, particularly for the fabrication of biosensor. Reza et al. [21] had fabricated tyrosinase biosensor based on rGO/Chit for electrochemical detection of bisphenol A. The reduced GO-AuNPs film for immobilization of tyrosinase for phenol detection has been studied [22]. Electrochemical biosensor for phenolic derivatives was also investigated using graphene–polyaniline and tyrosinase-modified GCE [23]. Furthermore, graphene/Co_3_O_4_ was used as platform for immobilization of tyrosinase and was successfully applied for catechol detection in fruit sample [24].

In this research, we have prepared Gr-Au-Chit-modified screen-printed carbon electrode (SPCE) immobilized with tyrosinase for the detection of phenol compounds in aqueous system. The constructed SPCE/Gr-Au-Chit exhibits an electrochemical advantage combination of Gr, Au and Chit., where the designed nanocomposite sensing material showed enhancement in analytical performances such as good selectivity, long-term stability and high sensitivity for phenolic compounds detection without the involvement of other electron mediators. Moreover, the developed biosensor was successfully applied for phenol detection in real samples.

## 2. Experimental

### 2.1. Reagents

Graphene powder was acquired from Graphenea (San Sebastian, Spain). HAuCl4, phenol, catechol, 4-nitrophenol, 4-chlorophenol and 2-4-Dichlorophenol were supplied from Sigma-Aldrich (St. Louis, MO, USA), Acros organics (Geel , Belgium), Merck ((Darmstadt, Germany) and Fluka (Buchs, Switzerland), respectively. Chitosan and tyrosinase enzyme from mushroom (EC1.14.18.1) were purchased from Sigma-Aldrich (St. Louis, MO, USA). Reagents were high in purity and used without further purification.

Phosphate buffer solution (PBS) was prepared from sodium phosphate dibasic (Na_2_HPO_4_) and sodium phosphate monobasic (NaH_2_PO_4_), which were obtained from Sigma-Aldrich (St. Louis, MO, USA). Phenolic standard solutions were prepared daily by dilution in PBS (0.1 M, pH 7.0).

### 2.2. Electrodes and Apparatus

Screen-printed carbon electrode (SPCE) was purchased from Rapid Lab Sdn Bhd (Bangi, Malaysia) which consists of carbon as working electrode (diameter: 5 mm), AgCl and carbon as reference, and counter electrodes, respectively. Electrochemical characterization and measurement were carried out using cyclic voltammetry, linear sweep voltammetry and differential pulse voltammetry by potentiostat (Autolab-PGSTAT, Utrecht, the Netherlands).

### 2.3. Preparation of Graphene-Gold-Chitosan/Tyrosinase Bionanocomposite

The synthesis of graphene-gold nanocomposite was according to previous literature [25] and the details are described briefly as follows: an aqueous solution of HAuCl_4_ was added to polyvinyl alcohol (PVA) solution with ratio of PVA/Au (wt/wt) of 0.65/1 with continuous stirring. Next, 0.1 M NaBH4 solution (NaBH_4_/Au (mol/mol) = 5/1) freshly prepared was then added to the above mixture under stirring until dark brown color sol formed. PVA acts as a protective ligand that prevents agglomeration of AuNPs, while NaBH_4_ acts as reducing agent for AuNPs [26]. Afterwards, 1.98 g of graphene was added to the mixture followed by adding a few drops of H_2_SO_4_ (acidified to pH 1–3, pH was checked using pH paper) under vigorous stirring conditions and the mixture was continuously stirred for 2 h. Finally, the prepared nanocomposite was filtered, washed thoroughly with 2 L of deionized water and dried at 120 °C in the oven for overnight.

Graphene-gold nanocomposite (Gr-Au) (1.5 mg/mL) was dispersed in deionized water and sonicated for 2 h at room temperature. Chitosan solution of 3 mg/mL was prepared by dissolving 6 mg of chitosan flake in 2.0 mL of 0.1 M acetic acid solution and then stirred for 3 h. Tyrosinase solution (6 mg/mL) was prepared in 0.1 M PBS (pH 7.0).

Gr-Au and chitosan solutions with a volume ratio of 3:1 were mixed and stirred for 30 min until homogeneous suspension was obtained. Next, 7 μL of the mixture (Gr-Au-Chit) was deposited onto the surface of working electrode, then left to dry at room temperature. Then, 5 μL of 6 mg/mL tyrosinase solution was applied onto the modified SPCE with Gr-Au-Chit and kept at 4 °C for drying purposes. The bioelectrode is referred to as SPCE/Gr-Au-Chit/Tyr. The enzyme electrode SPCE/Gr-Au-Chit/Tyr was then immersed in 0.1 M PBS (pH 7.0) to wash out the unbound enzyme. The biosensor was kept at 4 °C when not in use.

### 2.4. HPLC Analysis

High-performance liquid chromatography, HPLC (Water 2690) with UV-VIS detector was used for the chromatographic analysis of phenol in lake water sample. An Agilent Eclipse C18 column (250 mm × 4.6 mm, 5 μm) was used for sample separation. The mixture of 0.1% acetic acid solution and methanol (70:30, v/v) used as mobile phase. The flow rate, volume injection and wavelength were 1.0 mL/min, 10 μL and 270 nm, respectively.

## 3. Results and Discussion

### 3.1. Proposed Mechanism

**Scheme 1:**
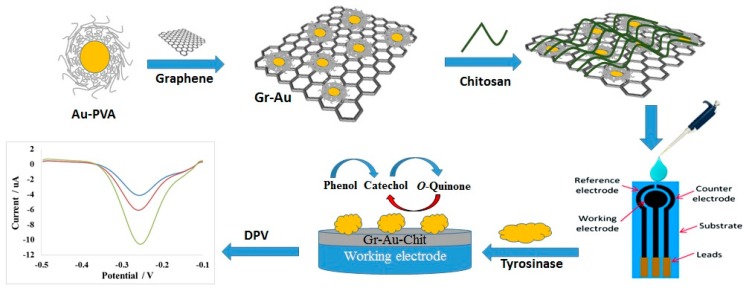
The procedure for the construction of the electrochemical biosensor. DPV = differential pulse voltammetry.

Scheme 1 illustrates the possible mechanism for the preparation of graphene composited with gold nanoparticles (AuNPs) and fabrication of electrochemical biosensor based on Gr-Au-Chit/Tyr. The synthesis strategy, first, gold nanoparticles in polyvinyl alcohol were formed on graphene nanosheets surface via hydrophilic interaction [27]. This is the important step to provide free-standing and stable Gr-Au dispersion for further assembly on the surface of electrode. The next step provided the electrostatic bonding between chitosan having a positive charge of amino groups –NH_3_^+^, and gold nanoparticles stabilized PVA with negative charge of the hydroxyl group –OH [28]. Finally, tyrosinase enzyme was immobilized onto the modified electrode via physical adsorption. The electrochemical signal observed was attributed to the reduction of *o*-quinone to catechol formed during enzymatic catalysis on the surface of electrode.

### 3.2. Characterization of the Modified Electrode

**Figure 1:**
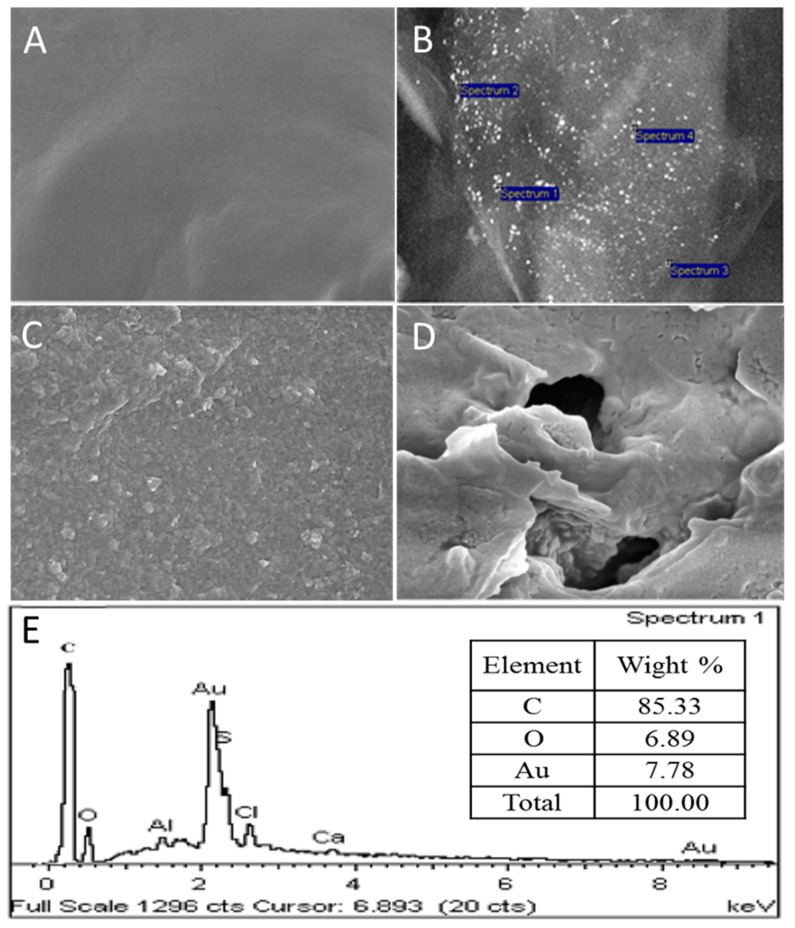
FESEM images of (**A**) Chitosan; (**B**) Gr-Au; (**C**) Gr-Au-Chit and (**D**) Gr-Au-Chit/Tyr; (**E**) EDX spectrum surface of Gr-Au-modified SPCE. EDX = Energy Dispersive X-Ray spectroscopy; SPCE = screen-printed carbon electrode.

The surface morphologies of the different modified SPCE were studied by Field emission scanning electron microscopy (FESEM). As shown in Figure 1A, the morphology of the chitosan film deposited on SPCE exhibited an excellent film-forming character with a homogenous and relatively smooth morphology. Figure 1B shows the FESEM image of Gr-Au nanocomposites, illustrating the position of bright dots of AuNPs over the entire surface, indicating that the AuNPs were linked on the graphene surface. After incorporating Gr-Au nanocomposite with chitosan solution, the morphology of Gr-Au-Chit film showed porous structure (Figure 1C). The reason for this observation was due to the interaction of negatively charged gold nanoparticles stabilized with PVA, with positive charge of chitosan, which result in the significant increase of the effective electrode surface for enzyme immobilization with good biocompatibility. It can be concluded that the Gr-Au-Chit had successfully dispersed on the SPCE surface. On the other hand, when tyrosinase enzyme was immobilized on the SPCE/Gr-Au-Chit surface, a homogeneous and significantly smoother morphology could be observed (Figure 1D), suggesting high coverage of the tyrosinase on the nanocomposite. Furthermore, Energy Dispersive X-Ray spectroscopy (EDX) provides the element analysis in the composite. Figure 1E confirmed the presence of gold in nanocomposite, indicating that the SPCE/Gr-Au was successfully prepared. The highest peak for Au was observed at 2.2 KeV.

**Figure 2:**
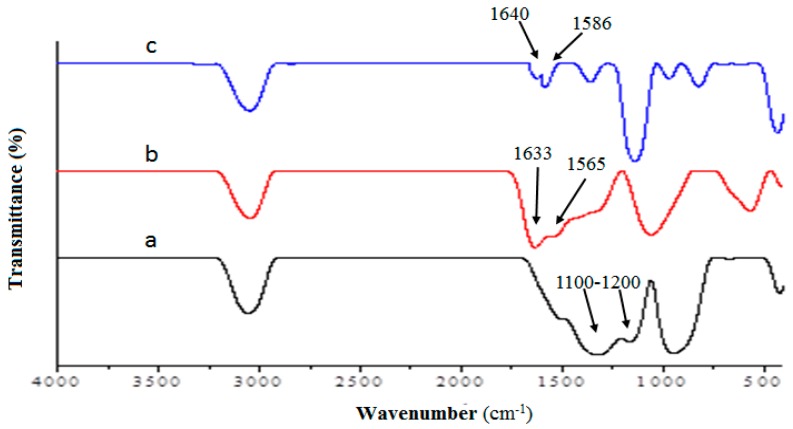
FTIR spectra of Gr-Au (**a**), Gr-Au-Chit (**b**), Gr-Au-Chit/Tyr (**c**).

The Fourier transform infrared spectroscopy (FTIR) spectra for Gr-Au, Gr-Au-Chit and Gr-Au-Chit/Tyr are shown in Figure 2 in the range of 400–4000 cm^−1^. Figure 2a displays broad peak between 1000 and 1200 cm^−1^, which could be attributed to oxygen functional groups on the defects of nanocomposite surface [29]. In the spectra of Gr-Au-Chit (Figure 2b), stretching bands of C=O appear at 1633 cm^−1^, while the bending vibration of amide groups N-H at 1565 cm^−1^ [28]. Moreover, when tyrosinase was immobilized on the Gr-Au-Chit film (Figure 2c), a resulting peak at 1640 cm^−1^ for amide I is due to –C=O stretching, while the peak at 1586 cm^−1^ is due to amide II as a result of C–N stretching, and –N–H bending and C–N stretching were observed [30,31]. The peaks at 1074 cm^−1^ and 3050 cm^−1^ might be caused by C–O vibration and O–H stretching, respectively.

**Figure 3:**
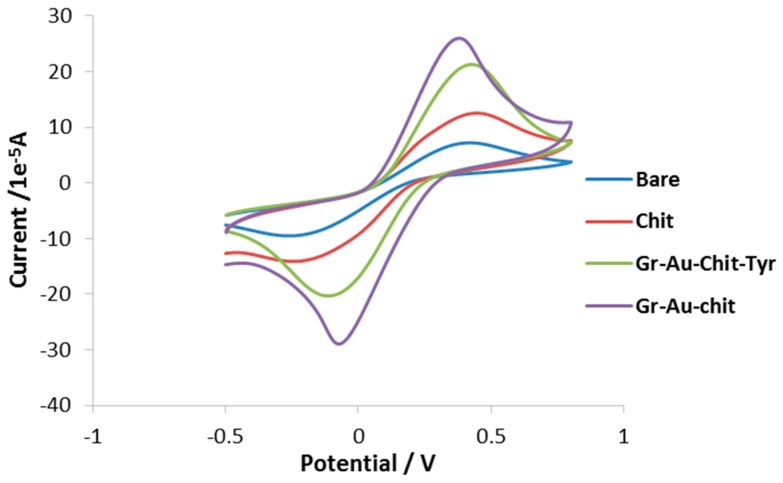
Cyclic voltammograms of the different modified SPCE in 5.0 mM K_3_Fe(CN)_6_/0.1 M KCl in the potential range of −0.5 to −0.8 V at scan rate 100 mV/s.

The electrochemical behaviour of the different modified electrodes was investigated by cyclic voltammetry. The peak-to-peak change of K_3_[Fe(CN)_6_] on the bare SPCE, SPCE/Chit, SPCE/Gr-Au-Chit and SPCE/Gr-Au-Chit/Tyr are shown in Figure 3. The bare SPCE exhibits electrochemical characteristics with a pair of redox peaks. After modification of SPCE with Chit, the electrochemical response gave an enhancement redox peak signal and the enhancement factor was calculated to be 1.78 times higher compared to bare SPCE. This behaviour could be attributed to the cationic nature of Chit [32,33]. A significant enhancement current was also observed at the SPCE/Gr-Au-Chit with calculated enhancement factor of 3.70 times higher compared to bare SPCE. This observation is due to the excellent conductivity and high surface area of graphene and the good distribution of AuNPs on graphene [34]. However, the redox peak current obtained with the SPCE/Gr-Au-Chit/Tyr is slightly lower than that of SPCE/Gr-Au-Chit, indicating that the immobilization of tyrosinase insulated the surface of electrode and hindered the electron transfer [21,35]. The oxidation peak potential (*Epa*) and reduction peak potential (*Epc*) are located at 0.35 and −0.05 V, respectively.

**Scheme 2:**
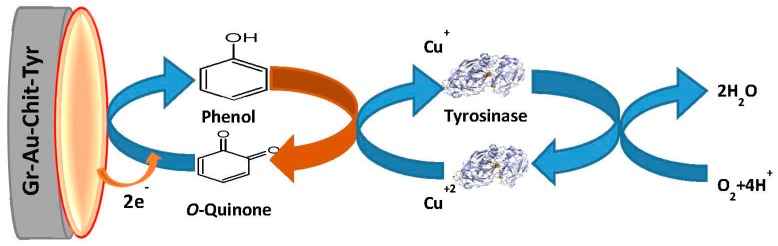
Possible mechanisms occur on the surface of electrode.

**Figure 4:**
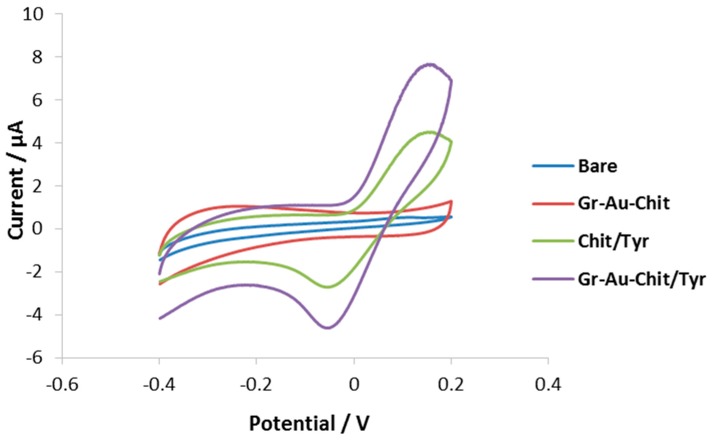
Cyclic voltammograms of phenol (0.5 × 10^−5^ M) at (a) bare SPCE, (b) SPCE/Gr-Au-Chit, (c) SPCE/Chit-Tyr and (d) SPCE/Gr-Au-Chit/Tyr. Scan rate of 100 mV/s and supporting electrolyte of 0.1 M PBS (pH 7.0) were used. PBS = Phosphate buffer solution.

Figure 4 displays the typical cyclic voltammograms of phenol in pH 7.0 (0.1 M PBS) at bare SPCE, SPCE/Gr-Au-Chit, SPCE/Chit/Tyr and SPCE/Gr-Au-Chit/Tyr. In the potential range between −0.4 to 0.2 V, no redox peak was observed at the bare SPCE and SPCE/Gr-Au-Chit in the absence of tyrosinase. It indicates that there is no catalytic activity for phenol for the respective electrodes. Instead, the reduction peak was observed at the potential of −0.08 V with the SPCE/Chit/Tyr and SPCE/Gr-Au-Chit/Tyr. In Figure 4, phenol reduction peak currents at SPCE/Gr-Au-Chit/Tyr are higher than SPCE/Chit/Tyr, which indicated that the Gr-Au nanocomposite plays a significant role in improvement of enzyme immobilization and facilitates the direct electron transfer between the substrate molecules and the modified electrode surface. The observed reduction peak is due to the reduction of *o*-quinone produced from the enzymatic reaction on the surface of electrode [36,37,38]. The mechanism of tyrosinase towards phenol has been reported previously [39]. Tyrosinase catalyses the oxidation of phenolic substrates in the presence of molecular oxygen to produce 1,2-dihydroxybenzene (catechol) and the subsequent oxidation of catechol to *o*-quinone [4,40]. The enzymatic reaction steps on the bioelectrode surface are shown in Scheme 2.

### 3.3. Optimization of the Experimental Parameters

**Figure 5:**
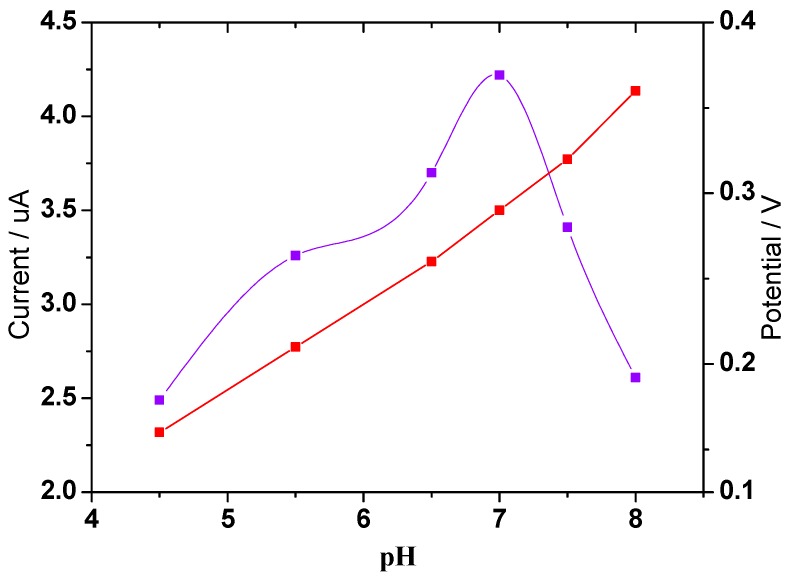
Effect of pH on the peak current and reduction potential of 5 μM of phenol at the surface of SPCE/Gr-Au-Chit/Tyr in the presence of 0.1 M PBS pH 7.0.

The influence of pH is a significant parameter in enzyme-based biosensor, which is correlated to the stability of biosensor and enzyme activity [41]. For that reason, influence of pH on the current response of Gr-Au-Chit/Tyr was studied in the pH range of 4.5–8.0. There is increasing in the reduction peak current with increasing of pH from 4.5 to 7.0, as shown in Figure 5. However, when the pH was further increased above 7, the reduction peak current decreased. The increasing of reduction peak with increasing pH is due to enzyme activity improvement. Thus, the hydroxyl groups in phenols reach the active site of enzyme in protonated state, then they deprotonated by the acid-base catalysis to bind with copper atoms of the binuclear centre of enzyme [42]. The decrease in peak current at pH > 7.0 is due to the involvement of proton in the reduction of *o*-quinone [43,44], which reacts with the hydroxyl group via hydrogen bonding, leading to provoking a bell-shaped curve at high pH values. The optimum biosensor response was achieved at pH 7.0 and the similar result was reported previously [45]. However, the tyrosinase activity would be decreased at higher or lower pH values [46]. Therefore, pH 7.0 (0.1 M PBS) was selected for further study.

Figure 5 also displays the relationship between pH and the reduction peak potential (*Epc*). It clearly indicates that the potential of peak current of Gr-Au-Chit/Tyr shifted to more negative with pH increasing from 4.5 to 8.0. The linear regression equation *Epc* (V) = 0.056 pH − 0.073 (R^2^ = 0.99). A shift of typically 56 mV per pH unit is in agreement with the theoretical value of 59 mV/pH [47,48], which indicates that the electron transfer in the electrode reaction was accompanied by the same number of protons.

The accumulation parameters effect was studied in the presence of phenol at fixed concentration (5 μM) in phosphate buffer solution. The reduction peak current increased with increased accumulation time up to 80 s. Subsequently, there was a slight decrease in the peak current with further increasing in accumulation time, meaning that the accumulation of phenol on Gr-Au-Chit/Tyr reached saturation. Furthermore, the effect of the accumulation potential on the reduction peak of phenol was also investigated in this study and the maximum reduction peak was achieved at −150 mV; a similar finding was observed by a previous researcher [49]. Thus, the accumulation steps in the experiment were fixed at potential of −150 mV for 80 s.

In order to achieve maximum response of the sensor, the effect of the amount of Gr-Au-Chit nanocomposite on the reduction peak current response of phenol (5 μM) was also evaluated. A series of different volumes of Gr-Au-Chit deposited on SPCE was studied. The current response increases upon raising the nanocomposite amount up to 7.0 μL and the response starts to decrease gradually when the amount increases above 8.0 μL. This might be due to the thickness of the nanocomposite film, which hindered the mass transfer and electron transfer on the surface of electrode [23,50]. Thus, an amount of 7.0 μL of the nanocomposite (Gr-Au-Chit) was used for fabrication of the bioelectrode.

**Figure 6:**
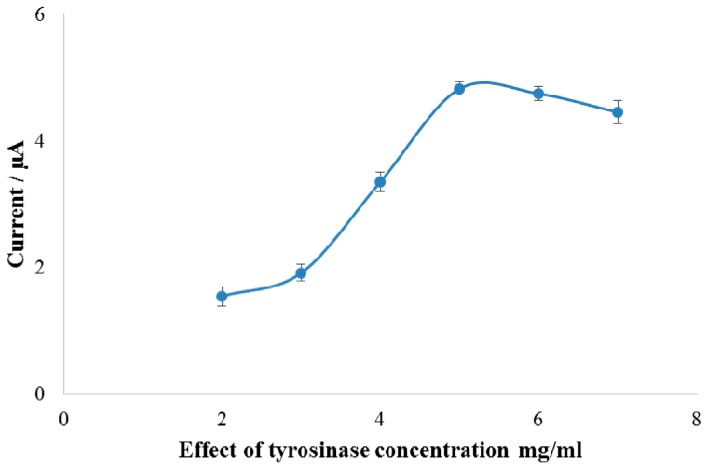
Effect of tyrosinase concentration (mg/mL) on the peak current of 5 μM of phenol in the presence of 0.1 M PBS (pH 7.0).

The amount of tyrosinase immobilized on the SPCE/Gr-Au-Chit surface affects the sensitivity of the developed sensor. Different concentrations of tyrosinase solution (2 to 7 mg/mL) were employed. In Figure 6, the reduction peak current was increased with increasing the concentration of tyrosinase from 2 to 5 mg/mL. Further increase caused a slightly decrease in the peak current response, which could possibly be related to the increased amount of tyrosinase increasing the resistance for interfacial electron transfer [51]. Therefore, an amount of 5 mg/mL of tyrosinase was applied for the fabrication of biosensor.

Linear sweep voltammetry (LSV) was used to study the scan rate effect on reduction peak of phenol in the range of 50–150 mV/s. A good linear correlation between the scan rate and reduction peak current was observed in the studied range. The regression equation was: *Ipc* (μA) = 0.043 v (mVs^‒1^) + 0.410; R^2^ = 0.99, which indicated that the electrochemical reduction process of Gr/Au/Chit/Tyr is a typical adsorption control. On the other hand, by plotting log (*Ipc*) versus log v, a straight line was obtained that can be expressed as: *Ipc* (μA) = 0.9112 logv (mVs^‒1^) − 0.1473; R^2^ = 0.99. The slope obtained of 0.91 is close to the theoretical value of 1.0, indicating that adsorption control process occurred.

### 3.4. Analytical Performance of the Developed Biosensor

**Figure 7:**
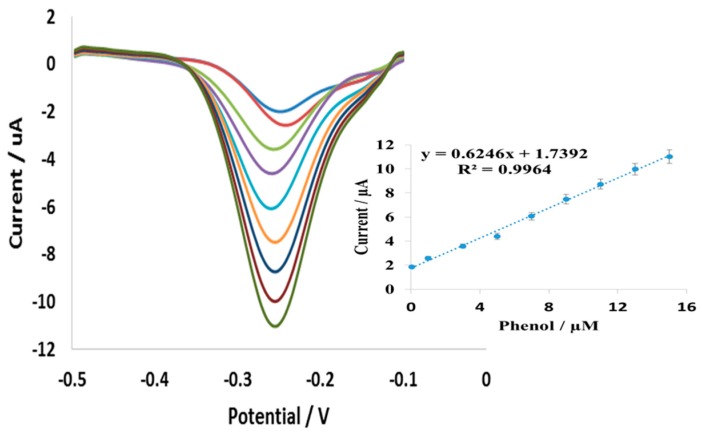
DPV obtained with the SPCE/Gr-Au-Chit/Tyr in 0.1 M PBS (pH 7.0) containing different concentrations of phenol from 0.05 μM to 15 μM. Inset is the calibration curve for the different concentrations of phenol in PBS. DPV = Differential pulse voltammetry.

Differential pulse voltammetry (DPV) technique was used to investigate the performance of the developed biosensor for the determination of phenol concentrations under optimum condition. Figure 7 shows that the reduction peak currents increased linearly with phenol concentrations in the range between 0.05 to 15.0 μM and the inset is the calibration curve of the SPCE/Gr-Au-Chit-Tyr. The linear regression equation was expressed as *i_p_*(μA) = 0.624C (μM) + 1.715 (R^2^ = 0.996), with the sensitivity of 0.624 μA μM^−1^ and the detection limit of 0.016 μM (S/N = 3), (where S is the standard deviation of blank (*n* = 3) and N is the slope of calibration curve).

The performance of the designed biosensor was also being tested with other phenolic compounds such as catechol, 4-chlorophenol, 4-nitrophenol and 2,4-dichlorophenol, respectively. Table 1 summarized the analytical performance of the proposed biosensor for the studied phenolic compounds including sensitivity, linear range, kinetic and detection limit; the sensitivity following the order of phenol > catechol > 4-chlorophenol > 4-nitrophenol > 2,4-dichlorophenol.

The apparent Michaelis–Menten constant value provides information on the enzyme activity for its substrate, where a lower value indicates a stronger substrate binding and higher catalytic activity. The values for this constant were 12.6, 13.1, 16.1, 17.8 and 24.2 μM for phenol, catechol, 4-chlorophenol, 4-nitrophenol and 2,4-dichlorophenol, respectively (Table 1).

The analytical performance of the developed biosensor was also compared with previous biosensor work for phenol determination. The K_m_ value of the present biosensor was lower than Tyr-PANI/GCE (85 μM) [52], Tyr-Au/GCE (140 μM) [51], Tyr-MWCNT-nafion/GCE (52 μM) [53], Tyr-PEDOT-rGO-Fe_2_O_3_/GCE (30 μM) [54] and Tyr/BiNP/SPE (84 μM) [39]. The low K_m_ values obtained indicated that the immobilized tyrosinase exhibited high affinity and high enzymatic activity towards phenolic substrates. The developed biosensor showed linear calibration curve towards phenol in the concentration range of 0.05–15 μM with sensitivity of 0.624 μA/μM and limit of detection of 0.016 μM, which is slightly better than previously reported works [43,55,56,57]. This result could be attributed to the efficient and high loading of tyrosinase on the Gr-Au-Chit nanocomposite film due to the large surface area and excellent conductivity of the nanocomposite.

The stability of the Gr-Au-Chit/Tyr-based biosensor was examined. After one month of storage at 4 °C under dried condition, it was found that the fabricated biosensor could retain approximately 85% of its original response. This relatively good stability might be attributed to the biocompatibility between tyrosinase and Gr-Au-Chit nanocomposite. The reproducibility and repeatability of biosensor was also investigated at phenol concentration of 2 μM and the relative standard deviation (SD) values obtained were 3.5% (*n* = 5) and 4.2% (*n* = 5), respectively.

### 3.5. Effect of Interfering Ions and Analytical Application of the Biosensor

To evaluate the sensor selectivity, effect of common interfering species for the determination of phenol was examined in the presence of interfering ion species. The experimental results of 100-fold concentration of K^+^, Mg^2+^, Ca^2+^, Fe^3+^, Zn^2+^, SO_4_^2−^, PO_4_^3−^, CO_3_^2−^, NO_3_^−^, 50-fold concentration of glucose and 10-fold excess concentration of ascorbic acid and uric acid did not show significant interfering influence on phenol determination, indicating that the proposed biosensor electrode showed high selectivity and great anti-interference ability.

### 3.6. Analysis of Real Sample

To demonstrate the practical application of the biosensor for phenol determination in water sample, the feasibility of the biosensor was tested. Water sample was collected from lake near University Putra Malaysia UPM area and was used for quantitative analysis. For unspiked water samples, no phenol was observed by the developed method and HPLC technique; these indicate that the sample did not have phenol. Therefore, the standard addition method was applied to investigate the performance of the proposed biosensor. Then, several known concentrations of phenol were spiked in lake water sample and measured using the developed biosensor and HPLC technique. As shown in Table 2, the critical values of |t| are higher than the calculated values, which indicates that no difference between the developed biosensor and HPLC methods with confidence level of 95% and the null hypothesis is accepted. According to the above result, the two methods used for phenol determination in spiked water samples are in good agreement and comparable.

## 4. Conclusions

The biosensor based on Gr-Au-Chit nanocomposite for phenolic compounds determination was successfully designed. The biosensor showed linear calibration curve towards phenol in the concentration range of 0.05–15 μM, sensitivity of 0.624 μA/μM with the limit of detection of 0.016 μM and good stability for at least one month. Other notable features of the biosensor include operation at room temperature, rapid response and stable operation. The proposed sensor provides good selectivity, reproducibility, and produced good recovery for real sample analysis. The SPCE/Gr-Au-Chit/Tyr also displays good performance, which was attributed to the excellent conductivity and large active surface area of graphene in combination with Au nanoparticles. Furthermore, the developed biosensor has shown potential applicability for monitoring of phenolic compounds in the environment.

## Figures and Tables

**Table 1:** Response characteristics of the tyrosinase biosensor towards phenolic compounds.

Phenolic Compound	Linear Range (μM)	Sensitivity (μA μM^−1^)	LOD (μM)	*K_m_* (μM)
Phenol	0.05–15	0.624	0.016	12.6
Catechol	0.05–11	0.483	0.02	13.1
4-chlorophenol	0.05–13	0.431	0.024	16.1
4-nitrophenol	0.05–19	0.334	0.031	17.8
2-4 Di chlorophenol	0.05–15	0.213	0.041	24.2

**Table 2:** Determination of phenol spiked in lake water sample by the proposed biosensor.

Sample	Added/μM	The Developed Method (μM) Mean ± SD (*n* = 3)	HPLC Method (μM) Mean ± SD (*n* = 3)	*t*-Test Value
Lake water	1.0	0.98 ± 0.0416	0.99 ± 0.0333	0.53
	1.5	1.46 ± 0.0503	1.53 ± 0.0727	1.42
	2.0	2.02 ± 0.0378	2.06 ± 0.0433	1.25
	2.5	2.39 ± 0.0305	2.43 ± 0.0338	1.05

Note: The critical value, t_4_ = 2.78 (*p* = 0.05).
